# The human lens is capable of trilineage differentiation towards osteo-, chondro-, and adipogenesis—a model for studying cataract pathogenesis

**DOI:** 10.3389/fbioe.2023.1164795

**Published:** 2023-05-31

**Authors:** Gerard Boix-Lemonche, Richard M. Nagymihaly, Xhevat Lumi, Goran Petrovski

**Affiliations:** ^1^ Department of Ophthalmology, Center for Eye Research and Innovative Diagnostics, Faculty of Medicine, Institute of Clinical Medicine, University of Oslo, Oslo, Norway; ^2^ Department of Ophthalmology, Oslo University Hospital, Oslo, Norway; ^3^ Eye Hospital, University Medical Centre Ljubljana, Ljubljana, Slovenia; ^4^ Faculty of Medicine, University of Ljubljana, Ljubljana, Slovenia; ^5^ Department of Ophthalmology, University of Split School of Medicine and University Hospital Centre, Split, Croatia

**Keywords:** human lens, trilineage differentiation, osteogenesis, chondrogenesis, adipogenesis, cataract

## Abstract

The potential for trilineage differentiation of cells in tissues represents a model for studying disease pathogenesis and regeneration pathways. Human lens trilineage differentiation has not yet been demonstrated, and so has calcification and osteogenic differentiation of human lens epithelial cells in the whole human lens. Such changes can pose a risk for complications during cataract surgery. Human lens capsules (*n* = 9) from cataract patients undergoing uneventful surgery were trilineage-differentiated toward osteogenesis, chondrogenesis, and adipogenesis. Furthermore, whole human healthy lenses (*n* = 3) collected from cadaveric eyes were differentiated into bone and characterized by immunohistochemistry. The cells in the human lens capsules were capable of undergoing trilineage differentiation, while the whole human healthy lenses could undergo osteogenesis differentiation, expressing osteocalcin, collagen I, and pigment epithelium-derived factor. We, hereby, show an *ex vivo* model for cataract formation through different stages of opacification, as well as provide *in vivo* evidence from patients undergoing calcified lens extraction with bone-like consistency.

## 1 Introduction

The human lens is a transparent biconvex structure located behind the iris of the eye, the sole purpose of which is to transmit light and focus rays onto the retina. The lens is made up of lens capsule, a membranous structure which encloses the lens matter. The latter consists of the superficial part or lens cortex made of nucleated cortical lens fiber cells (LFCs) and the central part, or lens nucleus, which is made of non-nucleated fiber cells. (Hejtmancik and Shiels, 2015) The inability of these cells to be replaced and the influence of nutritional, metabolic, and genetic factors make the lens susceptible to UV light and other environmental and oxidative stresses, resulting in loss of its transparency.(Quinlan and Clark, 2022) The human lens is surrounded by a collagenous capsule that acts as a barrier to diffusion and contributes to shaping the lens during natural focusing (accommodation). (Fisher and Pettet, 1972; Koretz and Handelman, 1988) The major structural molecules that self-assemble to form the matrix of the lens are type IV collagen, laminin, entactin, and perlecan. (Parmigiani and McAvoy, 1984; Cammarata et al., 1986) The lens also contains other molecules such as type XVIII collagen, heparin sulfate proteoglycans, and fibronectin. (Parmigiani and McAvoy, 1984; Cammarata et al., 1986) Approximately 2 billion people worldwide have moderate-to-severe vision impairment, of which 94 million cases are due to cataract formation. (Fricke et al., 2018; WHO, 2022) Cataract is the leading cause of blindness worldwide. (Asbell et al., 2005) By definition, it is an opacification of the crystalline lens, where the normal passage of light toward the retina may undergo visual axis opacification. (Lin et al., 2016) Specifically, the proteins contained in the human crystalline lens suffer certain alterations which increase its rigidity and eventually lead to loss of transparency. (Weinreb et al., 2001) Cataract surgery is one of the most frequently performed surgical procedures in ophthalmology. (Allen and Vasavada, 2006) Numerous researchers have worked on determining the etiology of cataracts, and several relevant mechanisms have been identified such as protein degradation, genetic variability, and oxidative stress. (Heiba et al., 1993; Beebe et al., 2010; Truscott and Friedrich, 2016; Wu et al., 2016) Although the regeneration potential of the lens could be shown^4^, the trilineage differentiation potential of the human lens has not yet been shown as doable. Some authors suggest that loss of elasticity and hardening of the lens is the major issue occurring with aging. (Glasser et al., 2001) Advanced cataracts have been shown to contain calcification, while osteogenic differentiation of lens epithelial cells (LECs) has been shown as a contributor in that process by us and collaborators. (Balogh et al., 2016) The environment and intercellular signals surrounding the lens can also affect the milieu and lead to a hardening of the lens and cataract development. It is well-known that LECs have the ability to react to environmental changes by undertaking an epithelial-to-mesenchymal transition (EMT) in reaction to inflammation, damage, or presence of certain growth factors. (Saika et al., 2003; Barbosa-Sabanero et al., 2012; Balogh et al., 2016; Lin et al., 2016) Endogenous stem/progenitor cells have been demonstrated to be a great alternative for tissue regeneration and tissue repair in the eye. (Enzmann et al., 2009) In this study, we generate a novel *ex vivo* human model for studying cataract formation based on the trilineage differential potential of the whole human lens. This might help better understand cataract pathogenesis and pathways for its inhibition or reversal.

## 2 Materials and methods

Human clear lenses (n = 4) were collected as leftover donor tissue for research purposes from the local cornea bank following the Guidelines of the Declaration of Helsinki and approval by the Regional Committees for Medical and Health Research Ethics, Norway (REK: 2017/418), while human lens capsules (n = 9) were collected from patients who had undergone cataract surgery at the Department of Ophthalmology, Oslo University Hospital, after patient informed consent was obtained.

For trilineage differentiation, osteogenesis, chondrogenesis, and adipogenesis kits were purchased (Gibco^®^, StemPro^®^, Thermo Fisher Scientific, MA, United States). Lens capsules were immersed in the culture media and cultured in differentiation media, while whole human lenses were cultured in osteogenesis differentiation media and Dulbecco’s modified Eagle’s medium/Nutrient Mixture F-12 (DMEM/F-12) with Glutamax™ supplemented with 10% fetal bovine serum (complete DMEM/F-12). This medium was replaced every 3–4 days for 2–4 weeks in a 12-well plate. At the end of the differentiation period, the medium was removed, and cells were fixed in 4% formalin solution. The fixed lens capsules were stained for markers of osteogenesis, chondrogenesis, and adipogenesis using Alizarin Red S (Merck/Sigma-Aldrich), Alcian Blue solution (Merck/Sigma-Aldrich), and HCS LipidTOX™ (Invitrogen, Thermo Fisher Scientific, MA, United States), respectively. Images were taken using a Zeiss upright light microscope and an EVOS FL fluorescent microscope.

For immunohistochemistry, paraffin-embedded sections fixed in 4% formalin from human lens tissue were analyzed by classical hematoxylin and eosin (H&E) and immune staining for osteocalcin (OCN; Proteintech, Manchester, Germany), collagen I (Thermo Fisher Scientific, MA, United States), and pigment epithelium-derived factor (PEDF; Thermo Fisher Scientific, MA, United States). Briefly, primary antibody incubation was carried out at room temperature (r.t.) for 30 min, secondary incubation at r. t. for 30 min, chromogen substrate with DAB Quanto (Thermo Fisher Scientific, MA, United States) incubation at r. t. for 10 min, counterstaining with H-E for contrast, and mounting with Pertex^®^ (Histolab^®^, Askim, Sweden) was performed. The results were visualized and recorded using an EVOS FL or Zeiss fluorescent microscope.

Presence of lens calcifications could be demonstrated in Grade 3 cataract from a patient who consented to use the surgical images accordingly.

## 3 Results

**FIGURE 1:**
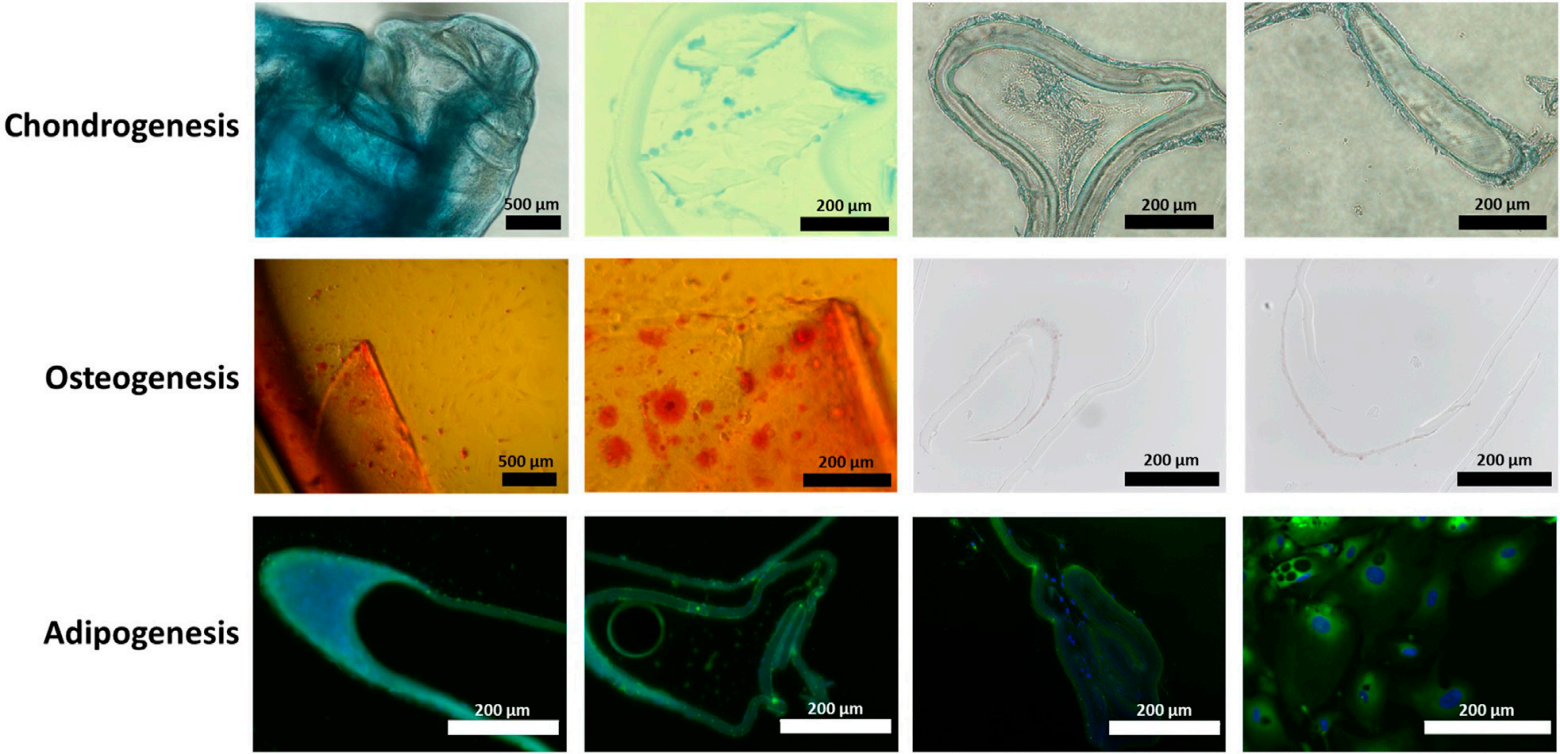
Human lens capsule trilineage differentiation. Human lens capsules immersed in **(A)** chondrogenic differentiation media for 21 days, **(B)** osteogenic differentiation media for 21 days, and **(C)** adipogenic differentiation media for 21 days. The scale bars of the first two images in chondro- and osteogenesis measure 500 μm, while the rest of the scale bars measure 200 µm.

Human LECs on human lens capsules were tested for their potential to undergo tri-lineage differentiation by culturing them in differentiation media for chondro-, osteo-, and adipogenesis for 21 days. At the end of the observation period, the lens capsules demonstrated ability for trilineage differentiation (Figure 1), confirmed by the presence of a chondrogenic pellet (Figure 1A), calcium deposits (Figure 1B), accumulation of neutral lipids (Figure 1C), and positive staining for markers of these lineages.

**FIGURE 2:**
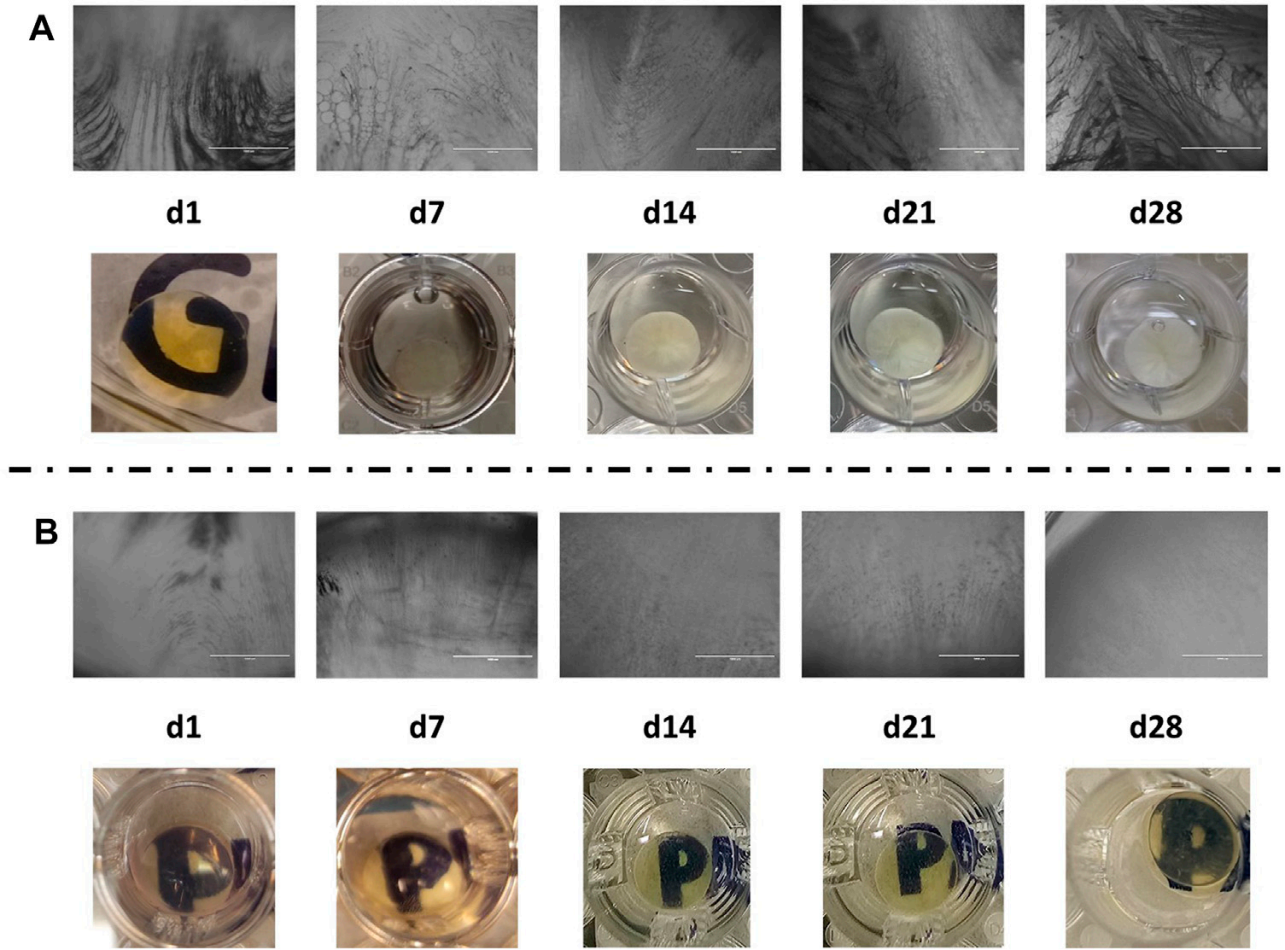
**(A)** Microscopic (aforementioned) and macroscopic (mentioned as follows) images of human lenses undergoing osteogenesis differentiation; **(B)** microscopic (aforementioned) and macroscopic (mentioned as follows) images of human lenses in complete DMEM/F-12.

To establish an *in vitro* model for cataract formation, we immersed whole human lenses in osteogenic differentiation medium for a 28-day period. After 7 days, the human lenses showed the presence of vacuoles, as well as opacification (Figure 2). These vacuoles were also observed at 14–28 days of incubation, specifically aggregation, in the center of the lens at day 28; the opacification of the lens in culture increased from cataract grade 1 (day 7) to 3–4 (day 28) (Figure 2) (Davis et al., 2010).

**FIGURE 3:**
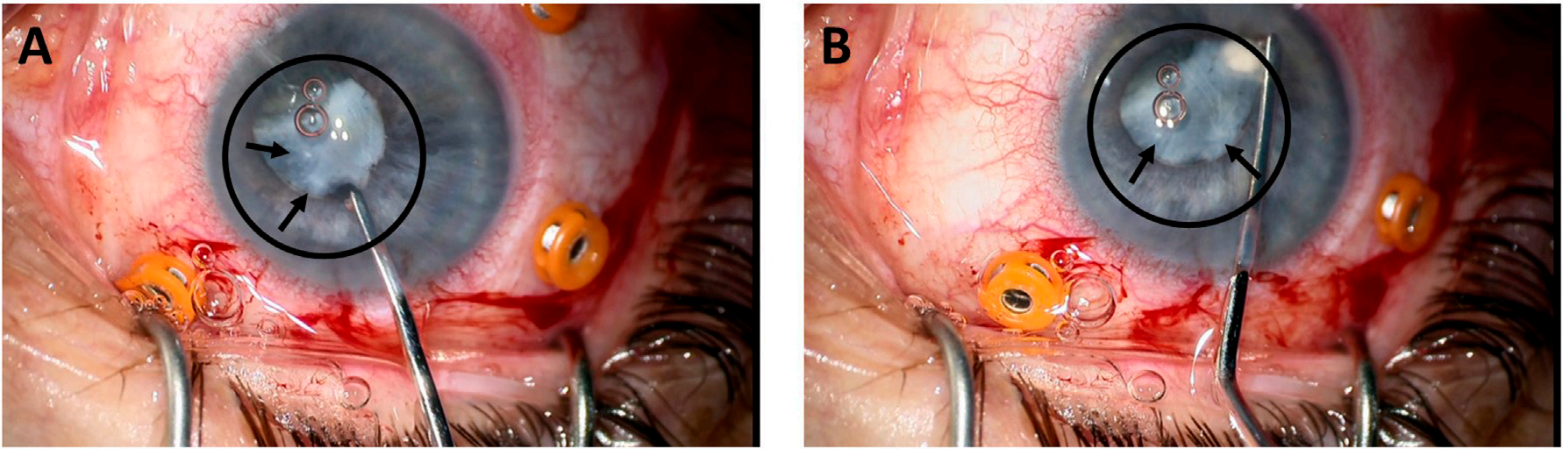
Removal of a grade 3 cataract from a patient **(A, B)**. The circle and the arrows indicate the position of the cataracted lens.

The presence of calcifications and lens opacification like that observed at day 28, under osteogenesis (Figure 2), is observed during grade 3 cataract extraction surgery as shown in Figure 3.

**FIGURE 4:**
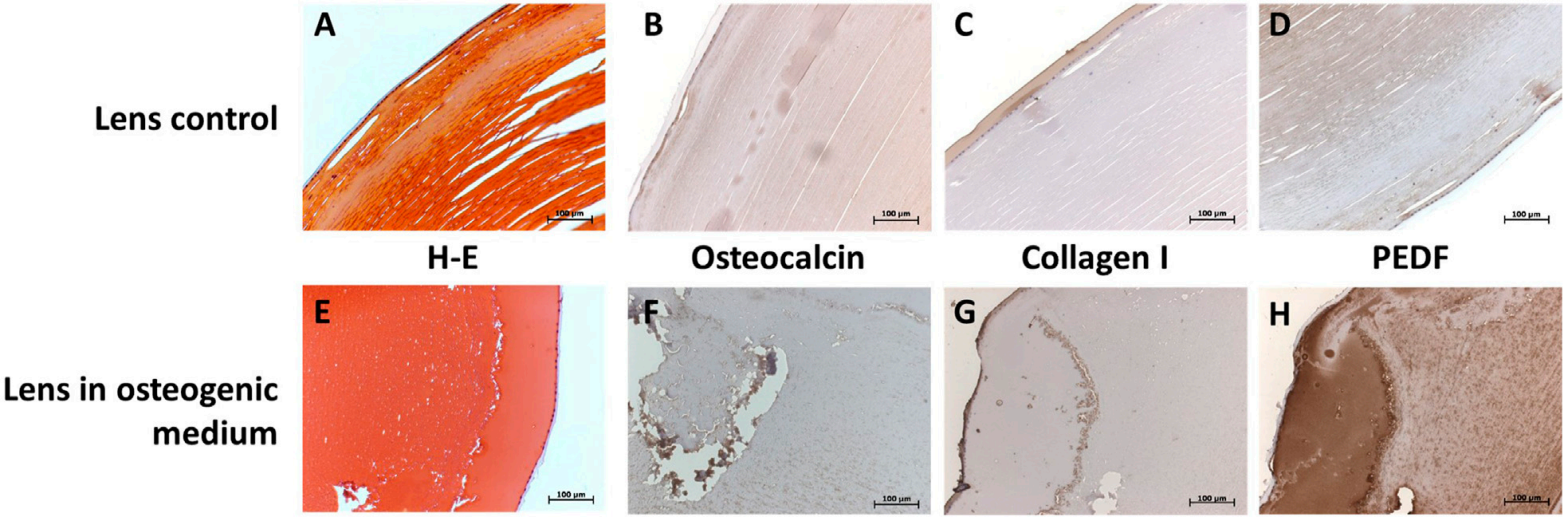
Immunohistological characterization of control human lenses (**A—D**; first row) and whole cultivated human lenses in osteogenic media for 28 days (**E—H**; second row) at 20X. Hematoxylin and eosin staining is shown in (**A**, **E**; first column), and staining for OCN (**B**, **F**; second column), collagen I (**C**, **G**; third column), and PEDF (**D**, **H**; fourth column). Presence of the protein is marked by the dark brown color.

The *in vitro* cataract formation after 28 days of cultivation of whole human lenses in osteogenic differentiation media showed positivity for known markers of this process: osteocalcin (OCN) (Figures 4B,F), collagen I (Figures 4C,G), and pigment epithelium-derived factor (PEDF) (Figures 4D,H).

## 4 Discussion

The differentiation of human lenses within their lens capsules is shown toward osteo-, chondro-, and adipogenesis, with the potential of modeling and, therefore, studying the process of cataract formation. Although not much is known about the adipogenic potential of the lens, pathological osteo- and chondrogenic differentiation of the LECs has been shown by us and collaborators (Balogh et al., 2016) as part of a process of ectopic calcification formation. Such ectopic calcification appears to be a well-regulated, cell-mediated process with many similarities to bone formation.

Presence of high concentrations of calcium and phosphate in bone-like hydroxyapatite crystals has been shown in senile cataracts, as well as congenital cataracts and chronic uveitis, like our findings, in grade 3–4 cataracts (Fagerholm et al., 1986; Chiang et al., 2004; Chen et al., 2005; Lin et al., 2010). Interestingly, opacification or calcification of artificial intraocular lenses has also been shown, further supporting the bone-like promoting environment surrounding the lens. (W. H. Shek et al., 2001; Yong et al., 2004)

Calcifying bone-forming benign tumors of the choroid, the innermost vascular layer of the eye, have been known, and factors implicated in their development include inflammation, trauma, hormonal state, calcium metabolism, environment, and heredity (Shields et al., 1988). In addition to osteogenesis, we have established a model for human lens chondro- and adipogenesis, the former having few examples in nature that are present in the eye. Namely, the distribution of scleral cartilage in vertebrates has been conserved across species. (Seko et al., 2008; Wu et al., 2015)

Orbital lipomas are benign adipose tissue tumors which contain lipoma-derived stem cells that have potential in regenerative medicine and tissue engineering due to their similar characteristics with adipose-derived stem cells (Stojanović et al., 2018).

Our model demonstrated, for the first time, that stimulation of whole human lenses and lens capsules containing LECs leads to osteogenic differentiation and ECM formation containing collagen I.

The environment surrounding the human lens contains calcium and inorganic phosphate as part of the aqueous humor, which seems to be enhanced in cataract patients (Kim and Choi, 2007). In diabetic subjects, the concentration of these constituents of the aqueous humor is even higher, thus representing higher risk for earlier cataract and calcification development (Balogh et al., 2016). The medium containing higher than normal concentration of calcium and inorganic phosphate or osteogenic compounds has previously been used by us and collaborators to trigger osteogenic differentiation of human LECs (Balogh et al., 2016); this likely contributes, in part, to the multifactorial process of osteogenic differentiation of these LECs, and of the whole lenses.

The progression of cataract is the result of changes in lens epithelial cells with deposition of aggregated proteins as a consequence of aging and oxidative damage, which causes clouding and loss of transparency in the crystalline lens. (Andley, 2009; Lim et al., 2020) The degree of lens opacification can be different and represents lens hardness or maturity level of cataract. (Mandelblum et al., 2020) Cataract develops and progresses through different stages, from early one or initial stages with relatively preserved transparency up to advanced stages, which, if not removed, reach the stage of mature or even hypermature cataract, which can be either white or completely dark and completely obscuring fundus view. (Michael and Bron, 2011) Until now, many efforts have been made by different authors and groups of experts to establish a comprehensive and internationally accepted classification that would represent clear gradations of the staging of cataracts. Such a gradation could be useful both for research purposes and in clinical practice, as it would provide clear information regarding the stage of cataract development and its prognosis and could make it easier for the surgeon to choose a proper surgical approach for removing it. In addition to the WHO Simplified Cataract Grading System, which grades three different forms of cataract (nuclear, cortical, and posterior subcapsular), the most widely used system is the Lens Opacities Classification System III (LOCS III). (Chylack, 1993; Thylefors et al., 2002) Other grading systems are BCN-10, the Japanese Cooperative Cataract Epidemiology Study Group System, the Standard Pre-Operative Nuclear Classification System (SPONCS), and many others that use different parameters and different grading schemes for standard types of cataracts. (Sasaki et al., 1990; Barraquer et al., 2017; Mandelblum et al., 2020) Aging of the crystalline lens and structural changes within the lens during cataract formation have been widely described. However, very little is known about the unusual processes of changing of the inner part of the lens in the form of osseous metaplasia. Intraocular osseous metaplasia has been mostly related to transdifferentiation of the retinal pigment epithelium and choroid. (Schnaudigel, 1989; Vemuganti et al., 2002; Munteanu et al., 2013) However, there are also case reports of the preserved potential of lens epithelial cells for osseous metaplasia as well. (Fledelius, 1975; Koinzer et al., 2009; Hadayer et al., 2018)

The potential of LECs toward proliferation, migration, and metaplasia is the main cause for one of the most common complications of cataract surgery, posterior capsule opacification (PCO). It is believed that the initial step for PCO development is the wound healing response of LEC to surgical trauma. (Cooksley et al., 2021) Through several mechanisms that involve inflammatory mediators, cytokines, and growth factors, LECs are activated to proliferate and to dedifferentiate into migratory spindle-like myofibroblasts. (Cooksley et al., 2021) Furthermore, these transformed cells are stimulated to secrete the extracellular matrix. (Cooksley et al., 2021) Both these steps lead to fibrosis, opacification, and contraction of the lens capsule. In addition to metaplasia and PCO formation, LECs have preserved the potential to regenerate, leading to pathological formations such are Soemmering’s ring and Elschnig’s pearls, which can obscure the visual axis and reduce the quality of vision in patients. (Cooksley et al., 2021).

Oxidative stress, due to aging and/or different external agents and factors, may break the homeostasis of the lens. (Micun et al., 2022) This could cause LECs to proliferate, adopting an alternative, migratory cell phenotype (epithelial–mesenchymal transition, EMT) and begin deposition of extracellular matrix components. (Saika et al., 1998; 2004; Joo et al., 1999) Immunohistology presented in this study showed staining for collagen I in the lens during osteogenic culturing (Figure 4). Presence of type I and several other types of collagen in cataractous lenses has been corroborated by other studies (Joo et al., 1999; Saika et al., 2004) and is an important marker of a fibrotic microenvironment. However, determining whether this collagen was deposited during cataract formation *in vivo* or during *ex vivo* culturing was not within the scope of the present study.

A substantial amount of PEDF was detected in the lens after osteogenic culturing (Figure 4). Conversely, in aging patients, a drop in intralenticular and intravitreal PEDF expression was described. (Segev et al., 2005; Yang et al., 2010; Yang and Zhang, 2015; Utlu et al., 2020) In the normal lens, PEDF is believed to be secreted by the LEC and is thought to inhibit neovascularization and apoptosis of the cells. (Ogata et al., 2002; Yang and Zhang, 2015) The role of the protein in cultured LECs is unclear, although it could speculatively be another marker of EMT (Alexander et al., 2020; Kuriyama et al., 2022), as a response to the culturing environment.

OCN expression is abundant in bone, cartilage, and dental tissues. It functions in the binding of hydroxyapatite in osteochondral tissues, as a mediator of mineralization, as a marker of bone metabolism (Balogh et al., 2016), and as a hormone (Lombardi et al., 2015). Its ectopic expression spans from calcified vessels and skin lesions (calcific uremic arteriolopathy) (Lian et al., 1983; Tyson et al., 2003) to endothelial progenitor cells (coronary artery disease) (Zhang et al., 2015). In our trilineage-differentiated human lenses, OCN likely functions in ectopic calcification, as osteogenic medium induces the expression of OCN in human lenses. As previously shown, control lenses do not express OCN, but cataractous lenses do (Balogh et al., 2016), which further supports our model for cataract formation.

Our findings strongly suggest that osteo-, chondro- and adipogenic stimuli induce-trilineage differentiation of human lenses and LECs, with the typical markers of such tissue formation being present.

We propose that this potential of the lens explains, to the least, the lens calcification formation in humans, as shown, provides a platform for *ex vivo* disease modeling.

## Data Availability

The original contributions presented in the study are included in the article/Supplementary Material; further inquiries can be directed to the corresponding authors.
